# Supplementary material for: Effect of Three-Dimensional-Printed Thermoplastics Used in Sensor Housings on Common Atmospheric Trace Gasses
Source: Sensors (Basel). 2024 Apr 19;24(8):2610. doi: 10.3390/s24082610 (PMC11053552; doi:10.3390/s24082610)
Supplement: Supplementary file 1 [file sensors-24-02610-s001.zip › sensors-2919151-supplementary.pdf]

# Supplementary Material: Effect of 3D-Printed Thermoplastics Used in Sensor Housings on Common Atmospheric Trace Gasses

Tristalee Mangin, Evan K. Blanchard, Kerry E. Kelly
Department of Chemical Engineering, University of Utah, Salt Lake City, UT 84112, USA

## Contents

**Figure S.1: Reaction Results in molar units** **3**

**Figure S.2: Chemical Structures for ABS, PC, PETG, PLA, and PVDF** **4**

**Figure S.3: Base plate design for the baffles** **5**

**Figure S.4: Vertical baffles design** **5**

**Table S.1: Off-gassing results for CO** **6**

**Table S.2: Off-gassing results for $CO_2$** **7**

**Table S.3: Off-gassing results for NO** **8**

**Table S.4: Off-gassing results for $NO_2$** **9**

**Table S.5: Off-gassing results for VOCs** **10**

**Table S.6: Reaction results for CO** **11**

**Table S.7: Reaction results for $CO_2$** **12**

**Table S.8: Reaction results for NO** **13**

**Table S.9: Reaction results for $NO_2$** **14**

**Table S.10: Reaction results for VOCs** **15**

**Table S.11: Significance testing results for VOC off-gassing rates** **16**

**Table S.12: NO and $NO_2$ kinetic equation** **16**

**Table S.13: NO and $NO_2$ kinetic equation in molar units** **17**

**Table S.14: FDM thermoplastic impact on trace gas concentrations** **18**

**Table S.15: Worst case FDM thermoplastic impact on trace gas concentrations** **19**

**Section S.1: FDM-printed Baffle Surface Area Calculation and Uncertainty Estimation** **20**

**Section S.2: Volumetric Flow Rate Uncertainty Calculation** **20**

**Section S.3: Equipment List** **20**

**Section S.4: VOC Off-gassing Results Null Hypothesis Significance Testing Calculation** **21**

**Section S.5: 3D FDM-printed Baffle Design and FDM Printer Settings** **22**

**Section S.6: Supplementary Material References** **22**

# Figure S.1

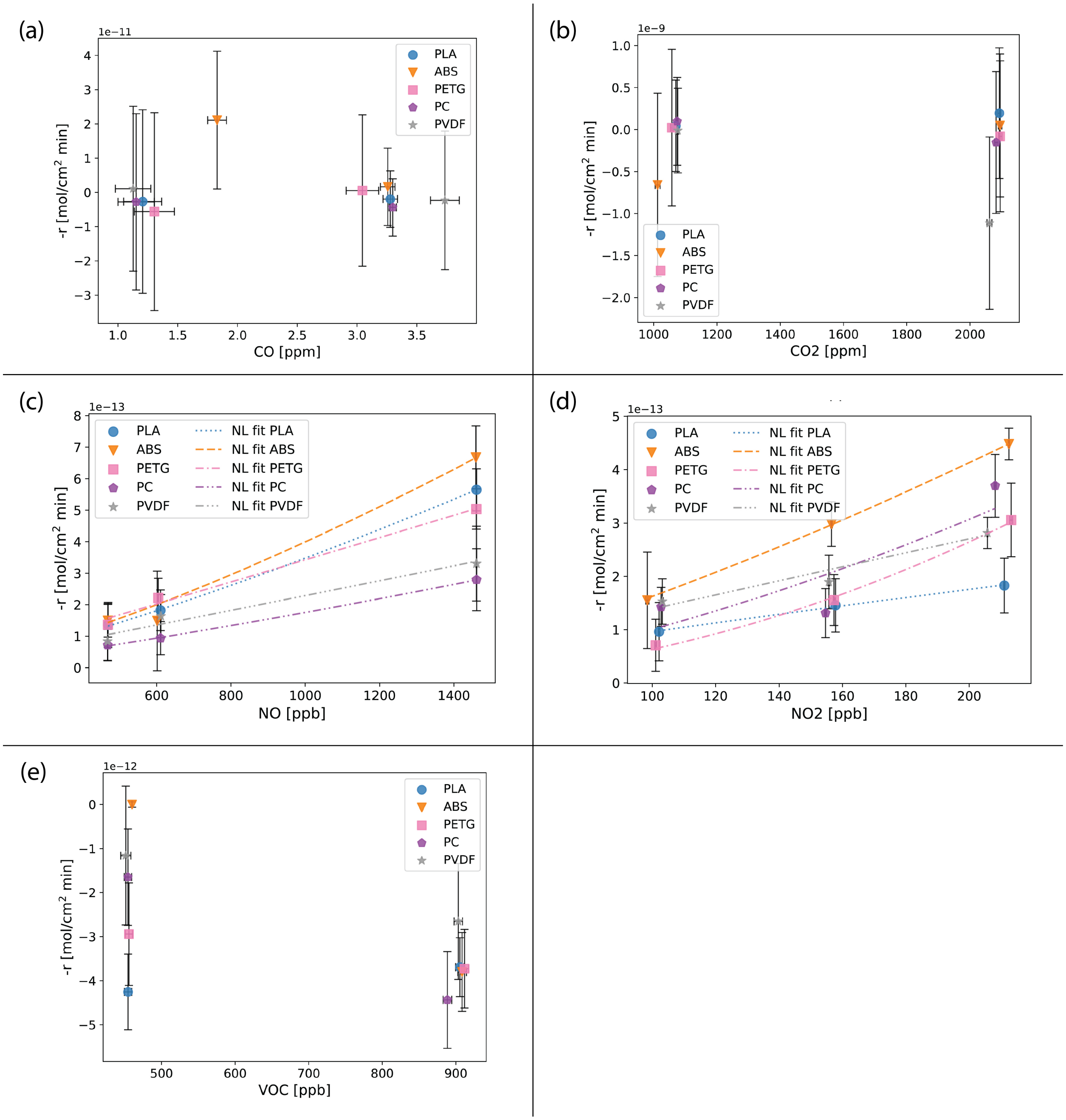


Figure S1: Reaction results converted to molar units for carbon monoxide (CO, a), carbon dioxide ($CO_2$, b), nitrogen monoxide (NO, c), nitrogen dioxide ($NO_2$, d), and volatile organic compounds (VOC, e) with the five thermoplastic materials - polylactic acid (PLA), acrylonitrile butadiene styrene (ABS), polyethylene terephthalate glycol (PETG), polycarbonate (PC), and polyvinylidene fluoride (PVDF).

# Figure S.2

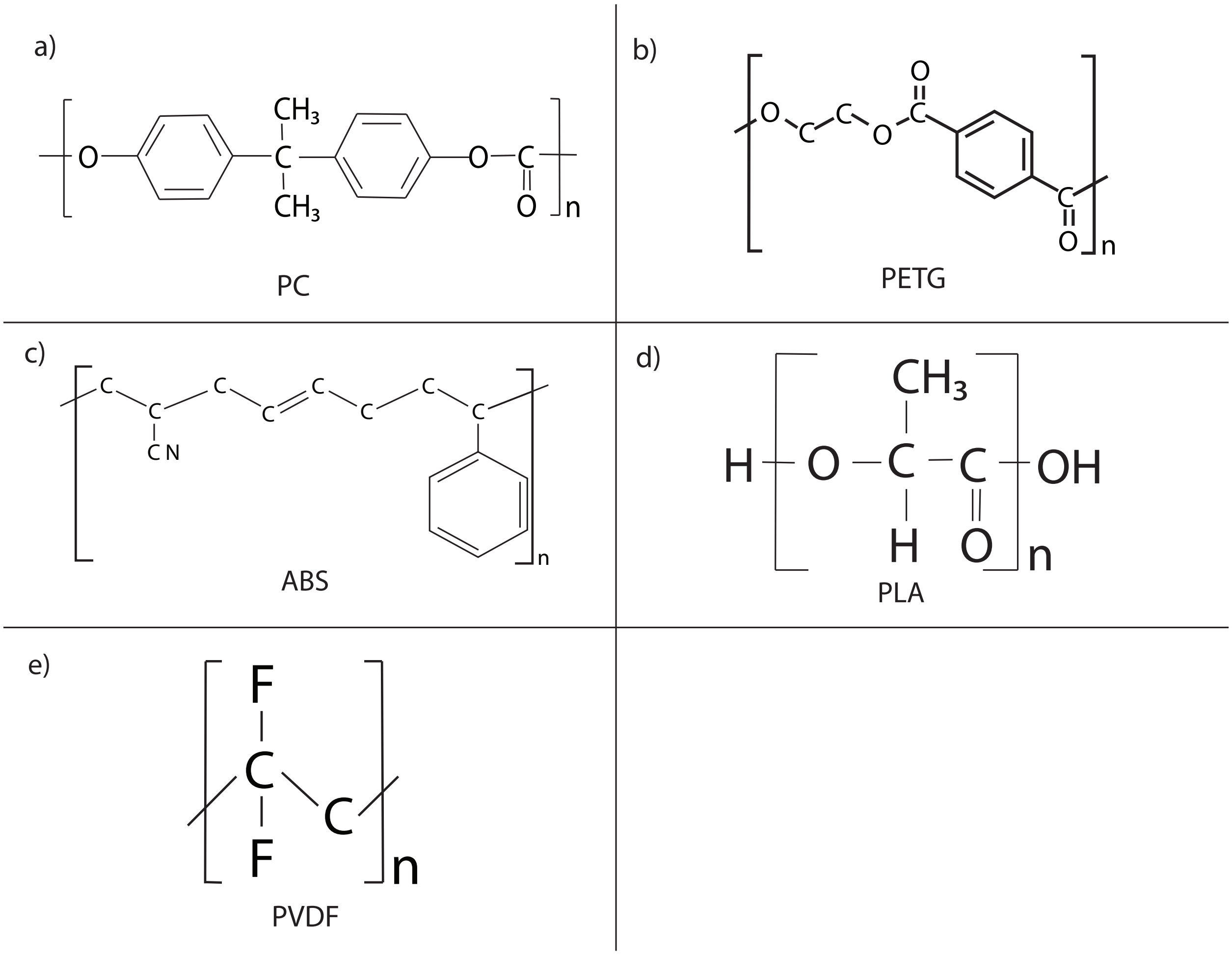


Figure S2: Chemical structures for polycarbonate (PC, a), polyethylene terephthalate glycol (PETG, b), acrylonitrile butadiene styrene (ABS, c), polylactic acid (PLA, d), and polyvinylidene fluoride (PVDF, e). The structures were adapted from references [12, 14, 13, 8, 4, 15, 11, 4, 3, 1, 9]. Pariskii et al.[10] listed polymer groups that are sensitive to $NO_2$. ABS contains nitrile groups and carbon-carbon double bonds[8, 4, 15] that are listed as sensitive to reactions with $NO_2$. However, PLA, PETG, PC, and PVDF do not have functional groups that are listed as sensitive to $NO_2$. PLA's main reactive group is the ester in the polymer backbone[11, 4, 3]. PETG contains carbonyl, ester, and aryl functional groups[14]. Aromatic PC polymer contains carbonate and aryl functional groups[12]. PVDF's main functional is a halogen[1, 9]

# Figure S.3

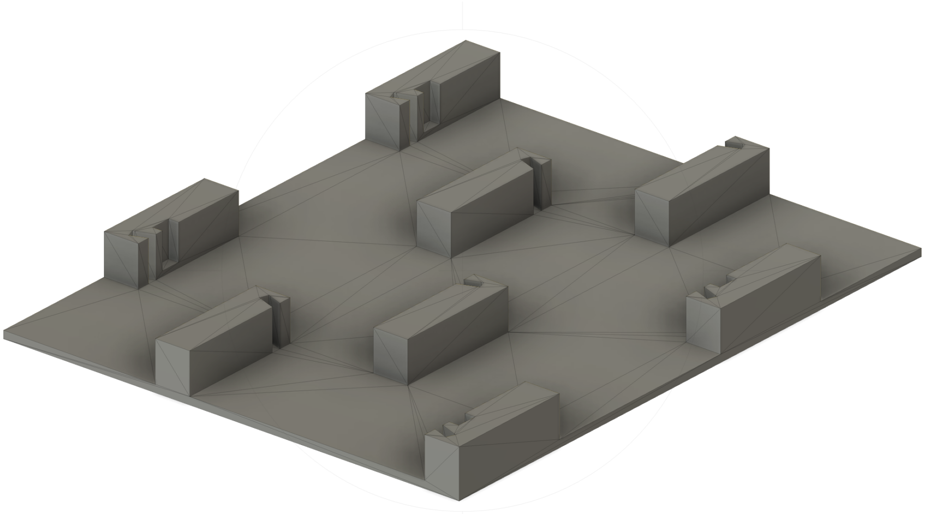

Figure S3: Screenshot of the baffle base plate designed in Fusion360.

# Figure S.4

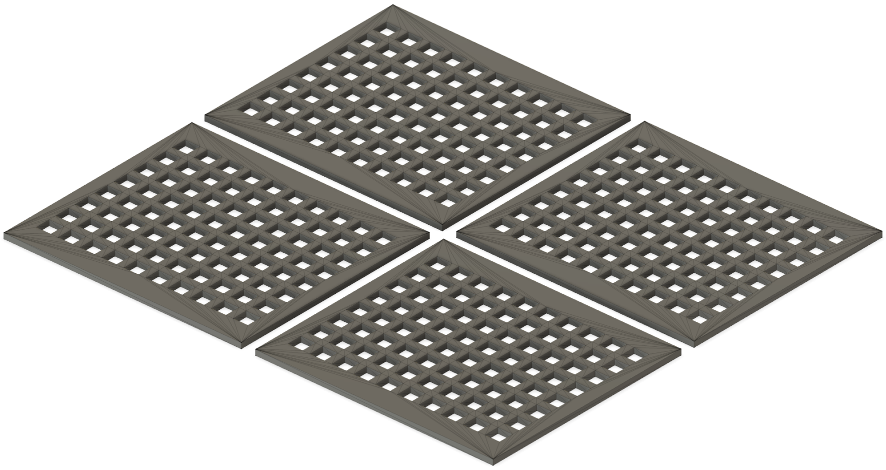

Figure S4: Screenshot of the vertical baffles designed in Fusion360.

# Table S.1

Table S1: Off-gassing results for carbon monoxide (CO) and each of the five thermoplastic materials - polylactic acid (PLA), acrylonitrile butadiene styrene (ABS), polyethylene terephthalate glycol (PETG), polycarbonate (PC), and polyvinylidene fluoride (PVDF). Off-gassing tests take place in a constant flow of zero air.

| Date | Gas (Unit) | Material | F (±std) | SA (±std) | $C_{SP}$ | $C_{A0}$ (±std) | $C_{A1}$ (±std) | ANOVA p-value | $-r_A$ (±error) | Molar$-r_A$ (±error) | T (±std) | RH (±std) |
|---|---|---|---|---|---|---|---|---|---|---|---|---|
| | | | (ccm) | (cm$^2$) | $(ppm)$ | $(ppm)$ | $(ppm)$ | | $\left(\frac{ccm*(ppm)}{cm^2}\right)$ | $\left(\frac{mol}{cm^2*min}\right)$ | (°C) | (%) |
| 6/12/23 | CO (ppm) | PLA | 1100 (±1.12) | 538.43 (±2.81) | 0 | 0.01 (±0.04) | 0.006 (±0.02) | 0.127 | 0 (±0.14) | $3.47x10^{-13}$ $(\pm5.82x10^{-12})$ | 24.2 (±0) | 10.52 (±0.23) |
| 6/12/23 | CO (ppm) | ABS | 1100 (±1.12) | 538.43 (±2.81) | 0 | 0 | 0.001 (±0.01) | 0.038 | 0 (±0.02) | $-1.20x10^{-13}$ $(\pm9.96x10^{-13})$ | 24.2 (±0) | 10.56 (±0.22) |
| 6/12/23 | CO (ppm) | PETG | 1100 (±1.12) | 538.43 (±2.81) | 0 | 0 | 0.002 (±0.01) | 0.007 | 0 (±0.03) | $-1.99x10^{-13}$ $(\pm1.28x10^{-12})$ | 24.42 (±0.04) | 10.67 (±0.31) |
| 6/12/23 | CO (ppm) | PC | 1100 (±1.12) | 538.43 (±5.62) | 0 | 0 | 0 | | 0 | 0 | 24.46 (±0.04) | 9.41 (±0.28) |
| 6/20/23 | CO (ppm) | PVDF | 1100 (±1.12) | 538.43 (±5.62) | 0 | 0.01 (±0.03) | 0.04 (±0.05) | <0.001 | -0.05 (±0.17) | $-2.18x10^{-12}$ $(\pm7.13x10^{-12})$ | 24.22 (±0.04) | 8.71 (±0.07) |

Column descriptions:
F - gas flowrate
SA - thermoplastic baffle surface area
$C_{SP}$ - dilution system gas concentration set point
$C_{A0}$ - baseline concentration measurements without the thermoplastic baffle in the acrylic chamber
$C_{A1}$ - concentration measurements with the thermoplastic baffle in the acrylic chamber
ANOVA p-value - analysis of variance (ANOVA) p-value if the $C_{A0}$ and $C_{A1}$ concentration measurements have different averages. A p-value of $<0.05$ is considered statistically significant in this study.
$-r_A$ - calculated reaction rate according to Equation 3
Molar $-r_A$ - calculated reaction rate according to Equation 3 converted to molar units
T - average experiment temperature
RH - average experiment relative humidity

# Table S.2

Table S2: Off-gassing results for carbon dioxide ($CO_2$) and each of the five thermoplastic materials - polylactic acid (PLA), acrylonitrile butadiene styrene (ABS), polyethylene terephthalate glycol (PETG), polycarbonate (PC), and polyvinylidene fluoride (PVDF). Off-gassing tests take place in a constant flow of zero air.

| Date | Gas (Unit) | Material | F (±std) | SA (±std) | $C_{SP}$ | $C_{A0}$ (±std) | $C_{A1}$ (±std) | ANOVA p-value | $-r_A$ (±error) | Molar$-r_A$ (±error) | T (±std) | RH (±std) |
|---|---|---|---|---|---|---|---|---|---|---|---|---|
| | | | (ccm) | (cm$^2$) | ($ppm$) | ($ppm$) | ($ppm$) | | $\left(\frac{ccm*(ppm)}{cm^2}\right)$ | $\left(\frac{mol}{cm^2*min}\right)$ | (°C) | (%) |
| 6/7/23 | $CO_2$ (ppm) | PLA | 1100 (±1.12) | 538.43 (±2.81) | 0 | 0 | 0 | N/A | 0 | 0 | 24.43 (±0.04) | 11.57 (±0.35) |
| 6/7/23 | $CO_2$ (ppm) | ABS | 1100 (±1.12) | 538.43 (±2.81) | 0 | 0 | 0 | N/A | 0 | 0 | 24.52 (±0.04) | 10.4 (±0.29) |
| 6/7/23 | $CO_2$ (ppm) | PETG | 1100 (±1.12) | 538.43 (±2.81) | 0 | 0 | 0 | N/A | 0 | 0 | 24.43 (±0.05) | 10.09 (±0.24) |
| 6/7/23 | $CO_2$ (ppm) | PC | 1100 (±1.12) | 538.43 (±5.62) | 0 | 0 | 0 | N/A | 0 | 0 | 24.84 (±0.07) | 9.61 (±0.23) |
| 6/21/23 | $CO_2$ (ppm) | PVDF | 1100 (±1.12) | 538.43 (±5.62) | 0 | 0 | 0 | N/A | 0 | 0 | 23.93 (±0.04) | 9.04 (±0.22) |

Column descriptions:
F - gas flowrate
SA - thermoplastic baffle surface area
$C_{SP}$ - dilution system gas concentration set point
$C_{A0}$ - baseline concentration measurements without the thermoplastic baffle in the acrylic chamber
$C_{A1}$ - concentration measurements with the thermoplastic baffle in the acrylic chamber
ANOVA p-value - analysis of variance (ANOVA) p-value if the $C_{A0}$ and $C_{A1}$ concentration measurements have different averages. A p-value of $<0.05$ is considered statistically significant in this study.
$-r_A$ - calculated reaction rate according to Equation 3
Molar $-r_A$ - calculated reaction rate according to Equation 3 converted to molar units
T - average experiment temperature
RH - average experiment relative humidity

# Table S.3

Table S3: Off-gassing results for nitrogen monoxide (NO) and each of the five thermoplastic materials - polylactic acid (PLA), acrylonitrile butadiene styrene (ABS), polyethylene terephthalate glycol (PETG), polycarbonate (PC), and polyvinylidene fluoride (PVDF). Off-gassing tests take place in a constant flow of zero air.

| Date | Gas (Unit) | Material | F (±std) | SA (±std) | $C_{SP}$ | $C_{A0}$ (±std) | $C_{A1}$ (±std) | ANOVA p-value | $-r_A$ (±error) | Molar$-r_A$ (±error) | T (±std) | RH (±std) |
|---|---|---|---|---|---|---|---|---|---|---|---|---|
| | | | (ccm) | (cm$^2$) | ($ppb$) | ($ppb$) | ($ppb$) | | $\left(\frac{ccm*(ppb)}{cm^2}\right)$ | $\left(\frac{mol}{cm^2*min}\right)$ | (°C) | (%) |
| 7/6/23 | NO (ppb) | PLA | 1100 (±1.12) | 538.43 (±2.81) | 0 | 0.15 (±0.04) | 0.18 (±0.03) | 0.534 | -0.05 (±0.16) | $-2.24x10^{-15}$ ($\pm 6.75x10^{-15}$) | 24.88 (±0.08) | 11.61 (±0.32) |
| 7/6/23 | NO (ppb) | ABS | 1100 (±1.12) | 538.43 (±2.81) | 0 | 0.10 (±0.05) | 0.15 (±0.06) | 0.143 | -0.1 (±0.26) | $-4.47x10^{-15}$ ($\pm 1.08x10^{-14}$) | 24.92 (±0.07) | 10.98 (±0.24) |
| 7/6/23 | NO (ppb) | PETG | 1100 (±1.12) | 538.43 (±2.81) | 0 | 0.07 (±0.05) | 0.06 (±0.03) | 0.780 | 0.01 (±0.18) | $7.62x10^{-16}$ ($\pm 7.60x10^{-15}$) | 25 (±0.01) | 10.76 (±0.18) |
| 7/6/23 | NO (ppb) | PC | 1100 (±1.12) | 538.43 (±5.62) | 0 | 0.04 (±0.02) | 0.03 (±0.03) | 0.503 | 0.02 (±0.13) | $1.07x10^{-15}$ ($\pm 5.51x10^{-15}$) | 24.8 (±0) | 8.83 (±0.05) |
| 7/6/23 | NO (ppb) | PVDF | 1100 (±1.12) | 538.43 (±5.62) | 0 | -0.03 (±0.01) | -0.15 (±0.05) | 0.022 | 0.25 (±0.12) | $1.03x10^{-14}$ ($\pm 5.26x10^{-15}$) | 25.26 (±0.07) | 8.82 (±0.14) |

Col-
umn descriptions:
F - gas flowrate
SA - thermoplastic baffle surface area
$C_{SP}$ - dilution system gas concentration set point
$C_{A0}$ - baseline concentration measurements without the thermoplastic baffle in the acrylic chamber
$C_{A1}$ - concentration measurements with the thermoplastic baffle in the acrylic chamber
ANOVA p-value - analysis of variance (ANOVA) p-value if the $C_{A0}$ and $C_{A1}$ concentration measurements have different averages. A p-value of $<0.05$ is considered statistically significant in this study.
$-r_A$ - calculated reaction rate according to Equation 3
Molar $-r_A$ - calculated reaction rate according to Equation 3 converted to molar units
T - average experiment temperature
RH - average experiment relative humidity

# Table S.4

Table S4: Off-gassing results for nitrogen dioxide ($NO_2$) and each of the five thermoplastic materials - polylactic acid (PLA), acrylonitrile butadiene styrene (ABS), polyethylene terephthalate glycol (PETG), polycarbonate (PC), and polyvinylidene fluoride (PVDF). Off-gassing tests take place in a constant flow of zero air.

| Date | Gas (Unit) | Material | F (±std) | SA (±std) | $C_{SP}$ | $C_{A0}$ (±std) | $C_{A1}$ (±std) | ANOVA p-value | $-r_A$ (±error) | Molar$-r_A$ (±error) | T (±std) | RH (±std) |
|---|---|---|---|---|---|---|---|---|---|---|---|---|
| | | | (ccm) | ($cm^2$) | ($ppb$) | ($ppb$) | ($ppb$) | | $\left(\frac{ccm*(ppb)}{cm^2}\right)$ | $\left(\frac{mol}{cm^2*min}\right)$ | (°C) | (%) |
| 6/22/23 | $NO_2$ (ppb) | PLA | 1100 (±1.12) | 538.43 (±2.81) | 0 | 1.51 (±0.03) | 1.61 (±0.06) | 0.025 | -0.2 (±0.2) | $-8.59x10^{-15}$ ($\pm 8.55x10^{-15}$) | 24.45 (±0.05) | 10.29 (±0.22) |
| 6/22/23 | $NO_2$ (ppb) | ABS | 1100 (±1.12) | 538.43 (±2.81) | 0 | 1.56 (±0.03) | 1.6 (±0.03) | 0.150 | -0.07 (±0.14) | $-3.05x10^{-15}$ ($\pm 5.83x10^{-15}$) | 24.5 (±0.02) | 9.85 (±0.23) |
| 6/22/23 | $NO_2$ (ppb) | PETG | 1100 (±1.12) | 538.43 (±2.81) | 0 | 1.56 (±0.03) | 1.58 (±0.04) | 0.543 | -0.03 (±0.16) | $-1.43x10^{-15}$ ($\pm 6.61x10^{-15}$) | 24.64 (±0.05) | 9.32 (±0.16) |
| 6/22/23 | $NO_2$ (ppb) | PC | 1100 (±1.12) | 538.43 (±5.62) | 0 | 1.51 (±0.08) | 1.57 (±0.08) | 0.178 | -0.12 (±0.35) | $-5.20x10^{-15}$ ($\pm 1.45x10^{-14}$) | 24.6 (±0.01) | 8.11 (±0.07) |
| 6/22/23 | $NO_2$ (ppb) | PVDF | 1100 (±1.12) | 538.43 (±5.62) | 0 | 1.48 (±0.03) | 1.52 (±0.04) | 0.181 | -0.07 (±0.14) | $-3.04x10^{-15}$ ($\pm 6.09x10^{-15}$) | 24.7 (±0.02) | 7.9 (±0.11) |

Column descriptions:
F - gas flowrate
SA - thermoplastic baffle surface area
$C_{SP}$ - dilution system gas concentration set point
$C_{A0}$ - baseline concentration measurements without the thermoplastic baffle in the acrylic chamber
$C_{A1}$ - concentration measurements with the thermoplastic baffle in the acrylic chamber
ANOVA p-value - analysis of variance (ANOVA) p-value if the $C_{A0}$ and $C_{A1}$ concentration measurements have different averages. A p-value of $<0.05$ is considered statistically significant in this study.
$-r_A$ - calculated reaction rate according to Equation 3
Molar $-r_A$ - calculated reaction rate according to Equation 3 converted to molar units
T - average experiment temperature
RH - average experiment relative humidity

# Table S.5

Table S5: Off-gassing results for volatile organic compounds (VOC) and each of the five thermoplastic materials - polylactic acid (PLA), acrylonitrile butadiene styrene (ABS), polyethylene terephthalate glycol (PETG), polycarbonate (PC), and polyvinylidene fluoride (PVDF). Off-gassing tests take place in a constant flow of zero air.

| Date | Gas (Unit) | Material | F (±std) | SA (±std) | $C_{SP}$ | $C_{A0}$ (±std) | $C_{A1}$ (±std) | ANOVA p-value | $-r_A$ (±error) | Molar$-r_A$ (±error) | T (±std) | RH (±std) |
|---|---|---|---|---|---|---|---|---|---|---|---|---|
| | | | (ccm) | (cm$^2$) | ($ppb$) | ($ppb$) | ($ppb$) | | $\left(\frac{ccm*(ppb)}{cm^2}\right)$ | $\left(\frac{mol}{cm^2*min}\right)$ | (°C) | (%) |
| 6/15/23 | VOC (ppb) | PLA | 1100 (±1.12) | 538.43 (±2.81) | 0 | 30 (±0) | 38.95 (±3.06) | <0.001 | -18.28 (±6.27) | $-7.50x10^{-13}$ ($\pm 2.57x10^{-13}$) | 24.01 (±0.03) | 10.86 (±0.2) |
| 6/15/23 | VOC (ppb) | ABS | 1100 (±1.12) | 538.43 (±2.81) | 0 | 25.33 (±2.5) | 40.38 (±1.91) | <0.001 | -30.74 (±9.03) | $-1.26x10^{-12}$ ($\pm 3.70x10^{-13}$) | 24.3 (±0.02) | 10.35 (±0.23) |
| 6/15/23 | VOC (ppb) | PETG | 1100 (±1.12) | 538.43 (±2.81) | 0 | 20.26 (±2.5) | 30 (±0) | <0.001 | -19.88 (±5.11) | $-8.15x10^{-13}$ ($\pm 2.10x10^{-13}$) | 24.36 (±0.04) | 10.15 (±0.22) |
| 6/15/23 | VOC (ppb) | PC | 1100 (±1.12) | 538.43 (±5.62) | 0 | 19.93 (±1.15) | 30.61 (±2.41) | <0.001 | -21.83 (±7.28) | $-8.95x10^{-13}$ ($\pm 2.99x10^{-13}$) | 24.64 (±0.05) | 9.04 (±0.16) |
| 6/15/23 | VOC (ppb) | PVDF | 1100 (±1.12) | 538.43 (±5.62) | 0 | 19.8 (±1.96) | 20 (±0) | 0.039 | -0.4 (±4.01) | $-1.67x10^{-14}$ ($\pm 1.65x10^{-13}$) | 24.43 (±0.04) | 7.88 (±0.06) |

Column descriptions:
F - gas flowrate
SA - thermoplastic baffle surface area
$C_{SP}$ - dilution system gas concentration set point
$C_{A0}$ - baseline concentration measurements without the thermoplastic baffle in the acrylic chamber
$C_{A1}$ - concentration measurements with the thermoplastic baffle in the acrylic chamber
ANOVA p-value - analysis of variance (ANOVA) p-value if the $C_{A0}$ and $C_{A1}$ concentration measurements have different averages. A p-value of <0.05 is considered statistically significant in this study.
$-r_A$ - calculated reaction rate according to Equation 3
Molar $-r_A$ - calculated reaction rate according to Equation 3 converted to molar units
T - average experiment temperature
RH - average experiment relative humidity

# Table S.6

Table S6: Reaction results for carbon monoxide (CO) and each of the five thermoplastic materials - polylactic acid (PLA), acrylonitrile butadiene styrene (ABS), polyethylene terephthalate glycol (PETG), polycarbonate (PC), and polyvinylidene fluoride (PVDF). Two gas concentration tests were run for each of the five thermoplastic materials. A third concentration was not run since no-to-limited reaction was observed between CO and the thermoplastic materials.

| Date | Gas (Unit) | Material | F (±std) | SA (±std) | $C_{SP}$ | $C_{A0}$ (±std) | $C_{A1}$ (±std) | ANOVA p-value | $-r_A$ (±error) | Molar$-r_A$ (±error) | T (±std) | RH (±std) |
|---|---|---|---|---|---|---|---|---|---|---|---|---|
| | | | (ccm) | (cm$^2$) | $(ppm)$ | $(ppm)$ | $(ppm)$ | | $\left(\frac{ccm*(ppm)}{cm^2}\right)$ | $\left(\frac{mol}{cm^2*min}\right)$ | (°C) | (%) |
| 7/12/23 | CO (ppm) | PLA | 1100 (±1.12) | 538.43 (±2.81) | 4 | 3.27 (±0.06) | 3.3 (±0.03) | <0.001 | -0.04 (±0.2) | $-1.95x10^{-12}$ ($\pm 8.25x10^{-12}$) | 24.47 (±0.04) | 10.48 (±0.23) |
| 8/29/23 | CO (ppm) | PLA | 1100 (±1.12) | 538.43 (±2.81) | 2 | 1.2 (±0.15) | 1.23 (±0.16) | 0.027 | -0.06 (±0.65) | $-2.67x10^{-12}$ ($\pm 2.68x10^{-11}$) | 24.1 (±0.08) | 12.94 (±0.25) |
| 7/12/23 | CO (ppm) | ABS | 1100 (±1.12) | 538.43 (±2.81) | 4 | 3.25 (±0.06) | 3.23 (±0.07) | 0.008 | 0.03 (±0.27) | $1.63x10^{-12}$ ($\pm 1.13x10^{-11}$) | 24.45 (±0.05) | 9.58 (±0.18) |
| 8/29/23 | CO (ppm) | ABS | 1100 (±1.12) | 538.43 (±2.81) | 2 | 1.82 (±0.08) | 1.58 (±0.16) | <0.001 | 0.51 (±0.49) | $2.11x10^{-11}$ ($\pm 2.01x10^{-11}$) | 23.59 (±0) | 13.47 (±0.28) |
| 7/12/23 | CO (ppm) | PETG | 1100 (±1.12) | 538.43 (±2.81) | 4 | 3.04 (±0.13) | 3.03 (±0.12) | 0.638 | 0.01 (±0.53) | $5.38x10^{-13}$ ($\pm 2.21x10^{-11}$) | 24.45 (±0.05) | 10.11 (±0.18) |
| 8/29/23 | CO (ppm) | PETG | 1100 (±1.12) | 538.43 (±2.81) | 2 | 1.3 (±0.16) | 1.37 (±0.17) | <0.001 | -0.13 (±0.7) | $-5.60x10^{-12}$ ($\pm 2.89x10^{-11}$) | 23.94 (±0.04) | 12.69 (±0.19) |
| 7/12/23 | CO (ppm) | PC | 1100 (±1.12) | 538.43 (±5.62) | 4 | 3.29 (±0.03) | 3.35 (±0.06) | <0.001 | -0.1 (±0.2) | $-4.38x10^{-12}$ ($\pm 8.37x10^{-12}$) | 24.47 (±0.04) | 9.63 (±0.16) |
| 8/29/23 | CO (ppm) | PC | 1100 (±1.12) | 538.43 (±5.62) | 2 | 1.15 (±0.15) | 1.18 (±0.15) | 0.018 | -0.06 (±0.62) | $-2.75x10^{\{} - 12\}$ ($\pm 2.57x10^{-11}$) | 24.07 (±0.06) | 12.11 (±0.22) |
| 6/20/23 | CO (ppm) | PVDF | 1100 (±1.12) | 538.43 (±5.62) | 4 | 3.73 (±0.12) | 3.76 (±0.12) | 0.123 | -0.05 (±0.49) | $-2.33x10^{\{} - 12\}$ ($\pm 2.02x10^{-11}$) | 24.96 (±0.07) | 8.47 (±0.22) |
| 8/29/23 | CO (ppm) | PVDF | 1100 (±1.12) | 538.43 (±5.62) | 2 | 1.12 (±0.14) | 1.11 (±0.13) | 0.320 | 0.02 (±0.58) | $1.07x10^{-12}$ ($\pm 2.41x10^{-11}$) | 24.2 (±0.01) | 10.81 (±0.12) |

Column descriptions:
F - gas flowrate
SA - thermoplastic baffle surface area
$C_{SP}$ - dilution system gas concentration set point
$C_{A0}$ - baseline concentration measurements without the thermoplastic baffle in the acrylic chamber
$C_{A1}$ - concentration measurements with the thermoplastic baffle in the acrylic chamber
ANOVA p-value - analysis of variance (ANOVA) p-value if the $C_{A0}$ and $C_{A1}$ concentration measurements have different averages. A p-value of <0.05 is considered statistically significant in this study.
$-r_A$ - calculated reaction rate according to Equation 3
Molar $-r_A$ - calculated reaction rate according to Equation 3 converted to molar units
T - average experiment temperature
RH - average experiment relative humidity

# Table S.7

Table S7: Reaction results for carbon dioxide ($CO_2$) and each of the five thermoplastic materials - polylactic acid (PLA), acrylonitrile butadiene styrene (ABS), polyethylene terephthalate glycol (PETG), polycarbonate (PC), and polyvinylidene fluoride (PVDF). Two gas concentration tests were run for each of the five thermoplastic materials. A third concentration was not run since no-to-limited reaction was observed between $CO_2$ and the thermoplastic materials.

| Date | Gas (Unit) | Material | F (±std) | SA (±std) | $C_{SP}$ | $C_{A0}$ (±std) | $C_{A1}$ (±std) | ANOVA p-value | $-r_A$ (±error) | Molar$-r_A$ (±error) | T (±std) | RH (±std) |
|---|---|---|---|---|---|---|---|---|---|---|---|---|
| | | | (ccm) | ($cm^2$) | (*ppm*) | (*ppm*) | (*ppm*) | | $\left(\frac{ccm*(ppm)}{cm^2}\right)$ | $\left(\frac{mol}{cm^2*min}\right)$ | (°C) | (%) |
| 6/7/23 | $CO_2$ (ppm) | PLA | 1100 (±1.12) | 538.43 (±2.81) | 2000 | 2092.54 (±4.87) | 2090.21 (±4.42) | <0.001 | 4.75 (±18.98) | $1.95x10^{-10}$ ($\pm 7.78x10^{-10}$) | 24.98 (±0.04) | 9.49 (±0.24) |
| 8/31/23 | $CO_2$ (ppm) | PLA | 1100 (±1.12) | 538.43 (±2.81) | 1000 | 1070.09 (±3.32) | 1069.55 (±3.17) | 0.461 | 1.09 (±13.27) | $4.50x10^{-11}$ ($\pm 5.44x10^{-10}$) | 24.69 (±0.06) | 12.57 (±0.42) |
| 6/7/23 | $CO_2$ (ppm) | ABS | 1100 (±1.12) | 538.43 (±2.81) | 2000 | 2094.87 (±5.5) | 2094.29 (±4.65) | 0.080 | 1.18 (±20.77) | $4.85x10^{-11}$ ($\pm 8.51x10^{-10}$) | 24.78 (±0.03) | 9.87 (±0.27) |
| 8/31/23 | $CO_2$ (ppm) | ABS | 1100 (±1.12) | 538.43 (±2.81) | 1000 | 1012.4 (±7.9) | 1020.28 (±5.13) | 0.142 | -16.08 (±26.62) | $-6.59x10^{-10}$ ($\pm 1.09x10^{-09}$) | 24 (±N/A) | 14 (±N/A) |
| 6/7/23 | $CO_2$ (ppm) | PETG | 1100 (±1.12) | 538.43 (±2.81) | 2000 | 2095.36 (±5.5) | 2096.32 (±5.21) | 0.005 | -1.95 (±21.9) | $-8.00x10^{-11}$ ($\pm 8.98x10^{-10}$) | 24.91 (±0.07) | 10.16 (±0.22) |
| 8/31/23 | $CO_2$ (ppm) | PETG | 1100 (±1.12) | 538.43 (±2.81) | 1000 | 1057.37 (±3.97) | 1057.1 (±7.15) | 0.879 | 0.55 (±22.74) | $2.28x10^{-11}$ ($\pm 9.32x10^{-10}$) | 24.52 (±0.04) | 14.3 (±0.73) |
| 6/7/23 | $CO_2$ (ppm) | PC | 1100 (±1.12) | 538.43 (±5.62) | 2000 | 2082.61 (±5.08) | 2084.45 (±4.99) | 0.001 | -3.76 (±20.59) | $-1.54x10^{-10}$ ($\pm 8.44x10^{-10}$) | 24.84 (±0.06) | 8.58 (±0.17) |
| 8/31/23 | $CO_2$ (ppm) | PC | 1100 (±1.12) | 538.43 (±5.62) | 1000 | 1074.16 (±3.16) | 1073 (±3.08) | 0.063 | 2.38 (±12.76) | $9.77x10^{-11}$ ($\pm 5.23x10^{-10}$) | 24.41 (±0.03) | 12.95 (±0.31) |
| 6/21/23 | $CO_2$ (ppm) | PVDF | 1100 (±1.12) | 538.43 (±5.62) | 2000 | 2061.55 (±7.71) | 2074.85 (±4.53) | <0.001 | -27.18 (±25.02) | $-1.11x10^{-9}$ ($\pm 1.03x10^{-09}$) | 24.4 (±0) | 8.36 (±0.15) |
| 8/31/23 | $CO_2$ (ppm) | PVDF | 1100 (±1.12) | 538.43 (±5.62) | 1000 | 1076.09 (±3.16) | 1076.23 (±2.85) | 0.793 | -0.3 (±12.29) | $-1.24x10^{-11}$ ($\pm 5.04x10^{-10}$) | 24.53 (±0.05) | 12.88 (±0.43) |

Column descriptions:
F - gas flowrate
SA - thermoplastic baffle surface area
$C_{SP}$ - dilution system gas concentration set point
$C_{A0}$ - baseline concentration measurements without the thermoplastic baffle in the acrylic chamber
$C_{A1}$ - concentration measurements with the thermoplastic baffle in the acrylic chamber
ANOVA p-value - analysis of variance (ANOVA) p-value if the $C_{A0}$ and $C_{A1}$ concentration measurements have different averages. A p-value of <0.05 is considered statistically significant in this study.
$-r_A$ - calculated reaction rate according to Equation 3
Molar $-r_A$ - calculated reaction rate according to Equation 3 converted to molar units
T - average experiment temperature
RH - average experiment relative humidity

# Table S.8

Table S8: Reaction results for nitrogen monoxide (NO) and each of the five thermoplastic materials - polylactic acid (PLA), acrylonitrile butadiene styrene (ABS), polyethylene terephthalate glycol (PETG), polycarbonate (PC), and polyvinylidene fluoride (PVDF). Three gas concentration tests were run for each of the five thermoplastic materials since the initial two gas concentration tests for NO showed statistically significant NO reaction rates.

| Date | Gas (Unit) | Material | F (±std) | SA (±std) | $C_{SP}$ | $C_{A0}$ (±std) | $C_{A1}$ (±std) | ANOVA p-value | $-r_A$ (±error) | Molar$-r_A$ (±error) | T (±std) | RH (±std) |
|---|---|---|---|---|---|---|---|---|---|---|---|---|
| | | | (ccm) | (cm$^2$) | ($ppb$) | ($ppb$) | ($ppb$) | | $\left(\frac{ccm*(ppb)}{cm^2}\right)$ | $\left(\frac{mol}{cm^2*min}\right)$ | (°C) | (%) |
| 7/6/23 | NO (ppb) | PLA | 1100 (±1.12) | 538.43 (±2.81) | 1000 | 610.39 (±0.36) | 608.22 (±0.4) | <0.001 | 4.44 (±1.57) | $1.82x10^{-13}$ ($\pm 6.46x10^{-14}$) | 25.23 (±0.04) | 9.82 (±0.14) |
| 9/12/23 | NO (ppb) | PLA | 1100 (±1.12) | 538.43 (±2.81) | 500 | 468.75 (±0.52) | 467.13 (±0.32) | <0.001 | 3.31 (±1.75) | $1.36x10^{-13}$ ($\pm 7.18x10^{-14}$) | 23.2 (±0) | 12.43 (±0.09) |
| 10/24/23 | NO (ppb) | PLA | 1100 (±1.12) | 538.43 (±2.81) | 1500 | 1460.8 (±0.56) | 1454.06 (±0.22) | <0.001 | 13.77 (±1.62) | $5.65x10^{-13}$ ($\pm 6.67x10^{-14}$) | 24 (±N/A) | 12 (±N/A) |
| 7/7/23 | NO (ppb) | ABS | 1100 (±1.12) | 538.43 (±2.81) | 1000 | 601.47 (±1.28) | 599.7 (±0.6) | 0.183 | 3.62 (±3.85) | $1.49x10^{-13}$ ($\pm 1.58x10^{-13}$) | 25.55 (±0.04) | 10.2 (±0.22) |
| 9/12/23 | NO (ppb) | ABS | 1100 (±1.12) | 538.43 (±2.81) | 500 | 468.32 (±0.33) | 466.54 (±0.28) | <0.001 | 3.64 (±1.26) | $1.49x10^{-13}$ ($\pm 5.19x10^{-14}$) | 23.5 (±0) | 11.98 (±0.07) |
| 10/24/23 | NO (ppb) | ABS | 1100 (±1.12) | 538.43 (±2.81) | 1500 | 1459.37 (±0.7) | 1451.4 (±0.48) | <0.001 | 16.28 (±2.42) | $6.67x10^{-13}$ ($\pm 9.96x10^{-14}$) | 23.84 (±0.04) | 12.78 (±0.17) |
| 7/7/23 | NO (ppb) | PETG | 1100 (±1.12) | 538.43 (±2.81) | 1000 | 604.28 (±0.39) | 601.64 (±0.34) | <0.001 | 5.4 (±1.52) | $2.21x10^{-13}$ ($\pm 6.24x10^{-14}$) | 25.9 (±0.08) | 10.63 (±0.2) |
| 9/12/23 | NO (ppb) | PETG | 1100 (±1.12) | 538.43 (±2.81) | 500 | 468.47 (±0.52) | 466.84 (±0.27) | <0.001 | 3.32 (±1.64) | $1.36x10^{-13}$ ($\pm 6.75x10^{-14}$) | 23.45 (±0.04) | 12.29 (±0.07) |
| 10/24/23 | NO (ppb) | PETG | 1100 (±1.12) | 538.43 (±2.81) | 1500 | 1460.44 (±0.36) | 1454.42 (±0.39) | <0.001 | 12.28 (±1.55) | $5.04x10^{-13}$ ($\pm 6.38x10^{-14}$) | 23.99 (±0.01) | 12.19 (±0.11) |
| 7/6/23 | NO (ppb) | PC | 1100 (±1.12) | 538.43 (±5.62) | 1000 | 610.62 (±0.36) | 609.5 (±0.25) | <0.001 | 2.28 (±1.28) | $9.37x10^{-14}$ ($\pm 5.26x10^{-14}$) | 25.28 (±0.06) | 9.19 (±0.13) |
| 9/12/23 | NO (ppb) | PC | 1100 (±1.12) | 538.43 (±5.62) | 500 | 467.89 (±0.19) | 467.03 (±0.39) | <0.001 | 1.74 (±1.21) | $7.17x10^{-14}$ ($\pm 4.98x10^{-14}$) | 23.72 (±0.08) | 11.6 (±0.1) |
| 10/24/23 | NO (ppb) | PC | 1100 (±1.12) | 538.43 (±5.62) | 1500 | 1461 (±0.56) | 1457.67 (±0.6) | <0.001 | 6.81 (±2.39) | $2.79x10^{-13}$ ($\pm 9.82x10^{-14}$) | 24.12 (±0.05) | 11.15 (±0.09) |
| 7/6/23 | NO (ppb) | PVDF | 1100 (±1.12) | 538.43 (±5.62) | 1000 | 611.34 (±0.34) | 609.38 (±0.46) | <0.001 | 3.99 (±1.66) | $1.64x10^{-13}$ ($\pm 6.81x10^{-14}$) | 25.38 (±0.07) | 8.72 (±0.14) |
| 9/12/23 | NO (ppb) | PVDF | 1100 (±1.12) | 538.43 (±5.62) | 500 | 467.5 (±0.26) | 466.49 (±0.46) | <0.001 | 2.04 (±1.49) | $8.40x10^{-14}$ ($\pm 6.11x10^{-14}$) | 23.6 (±0.02) | 11.06 (±0.07) |
| 10/24/23 | NO (ppb) | PVDF | 1100 (±1.12) | 538.43 (±5.62) | 1500 | 1460.78 (±0.82) | 1456.83 (±0.58) | <0.001 | 8.05 (±2.89) | $3.30x10^{-13}$ ($\pm 1.19x10^{-13}$) | 24.24 (±0.06) | 10.6 (±0.11) |

Column descriptions:
F - gas flowrate
SA - thermoplastic baffle surface area
$C_{SP}$ - dilution system gas concentration set point
$C_{A0}$ - baseline concentration measurements without the thermoplastic baffle in the acrylic chamber
$C_{A1}$ - concentration measurements with the thermoplastic baffle in the acrylic chamber
ANOVA p-value - analysis of variance (ANOVA) p-value if the $C_{A0}$ and $C_{A1}$ concentration measurements have different averages. A p-value of <0.05 is considered statistically significant in this study.
$-r_A$ - calculated reaction rate according to Equation 3
Molar $-r_A$ - calculated reaction rate according to Equation 3 converted to molar units
T - average experiment temperature
RH - average experiment relative humidity

# Table S.9

Table S9: Reaction results for nitrogen dioxide ($NO_2$) and each of the five thermoplastic materials - polylactic acid (PLA), acrylonitrile butadiene styrene (ABS), polyethylene terephthalate glycol (PETG), polycarbonate (PC), and polyvinylidene fluoride (PVDF). Three gas concentration tests were run for each of the five thermoplastic materials since the initial two gas concentration tests for $NO_2$ showed statistically significant $NO_2$ reaction rates.

| Date | Gas (Unit) | Material | F (±std) | SA (±std) | $C_{SP}$ | $C_{A0}$ (±std) | $C_{A1}$ (±std) | ANOVA p-value | $-r_A$ (±error) | Molar$-r_A$ (±error) | T (±std) | RH (±std) |
|---|---|---|---|---|---|---|---|---|---|---|---|---|
| | | | (ccm) | ($cm^2$) | ($ppb$) | ($ppb$) | ($ppb$) | | $\left(\frac{ccm*(ppb)}{cm^2}\right)$ | $\left(\frac{mol}{cm^2*min}\right)$ | (°C) | (%) |
| 6/22/23 | $NO_2$ (ppb) | PLA | 1100 (±1.12) | 538.43 (±2.81) | 200 | 211.08 (±0.2) | 208.9 (±0.41) | <0.001 | 4.46 (±1.25) | $1.83x10^{-13}$ ($\pm 5.13x10^{-14}$) | 25 (±0) | 9.2 (±0.17) |
| 9/14/23 | $NO_2$ (ppb) | PLA | 1100 (±1.12) | 538.43 (±2.81) | 100 | 102.15 (±0.25) | 101 (±0.4) | <0.001 | 2.34 (±1.33) | $9.62x10^{-14}$ ($\pm 5.46x10^{-14}$) | 24.02 (±0.06) | 13.55 (±0.35) |
| 10/26/23 | $NO_2$ (ppb) | PLA | 1100 (±1.12) | 538.43 (±2.81) | 150 | 152.25 (±0.19) | 149.96 (±0.3) | 0.009 | 4.68 (±1.02) | $1.92x10^{-13}$ ($\pm 4.21x10^{-14}$) | 24 (±N/A) | 14 (±N/A) |
| 6/22/23 | $NO_2$ (ppb) | ABS | 1100 (±1.12) | 538.43 (±2.81) | 200 | 212.54 (±0.2) | 207.19 (±0.14) | <0.001 | 10.92 (±0.71) | $4.48x10^{-13}$ ($\pm 2.94x10^{-14}$) | 25.25 (±0.06) | 9.48 (±0.23) |
| 9/14/23 | $NO_2$ (ppb) | ABS | 1100 (±1.12) | 538.43 (±2.81) | 100 | 98.44 (±0.49) | 96.58 (±0.58) | 0.054 | 3.78 (±2.2) | $1.55x10^{-13}$ ($\pm 9.05x10^{-14}$) | 23.81 (±0.07) | 13.94 (±0.41) |
| 10/26/23 | $NO_2$ (ppb) | ABS | 1100 (±1.12) | 538.43 (±2.81) | 150 | 143.64 (±0.37) | 142.98 (±0.53) | 0.729 | 1.34 (±1.85) | $5.53x10^{-14}$ ($\pm 7.61x10^{-14}$) | 24 (±N/A) | 14 (±N/A) |
| 6/22/23 | $NO_2$ (ppb) | PETG | 1100 (±1.12) | 538.43 (±2.81) | 200 | 213.25 (±0.46) | 209.6 (±0.36) | <0.001 | 7.45 (±1.67) | $3.06x10^{-13}$ ($\pm 6.88x10^{-14}$) | 25.28 (±0.06) | 8.99 (±0.19) |
| 9/14/23 | $NO_2$ (ppb) | PETG | 1100 (±1.12) | 538.43 (±2.81) | 100 | 101.07 (±0.29) | 100.23 (±0.28) | 0.038 | 1.72 (±1.19) | $7.07x10^{-14}$ ($\pm 4.88x10^{-14}$) | 23.91 (±0.05) | 14.62 (±5.82) |
| 10/26/23 | $NO_2$ (ppb) | PETG | 1100 (±1.12) | 538.43 (±2.81) | 150 | 149.22 (±0.15) | 147.83 (±0.41) | 0.048 | 2.83 (±1.18) | $1.16x10^{-13}$ ($\pm 4.85x10^{-14}$) | 24 (±N/A) | 14 (±N/A) |
| 6/22/23 | $NO_2$ (ppb) | PC | 1100 (±1.12) | 538.43 (±5.62) | 200 | 208.2 (±0.33) | 203.79 (±0.36) | <0.001 | 9.01 (±1.43) | $3.70x10^{-13}$ ($\pm 5.88x10^{-14}$) | 25.23 (±0.07) | 8.74 (±1.39) |
| 9/14/23 | $NO_2$ (ppb) | PC | 1100 (±1.12) | 538.43 (±5.62) | 100 | 102.79 (±0.29) | 101.08 (±0.14) | <0.001 | 3.48 (±0.89) | $1.43x10^{-13}$ ($\pm 3.67x10^{-14}$) | 24.17 (±0.07) | 11.79 (±1.91) |
| 10/26/23 | $NO_2$ (ppb) | PC | 1100 (±1.12) | 538.43 (±5.62) | 150 | 154.63 (±0.3) | 153.07 (±0.24) | <0.001 | 3.2 (±1.12) | $1.31x10^{-13}$ ($\pm 4.60x10^{-14}$) | 24 (±N/A) | 14 (±N/A) |
| 1/19/24 | $NO_2$ (ppb) | PVDF | 1100 (±1.12) | 538.43 (±5.62) | 200 | 205.76 (±0.14) | 202.39 (±0.21) | 0.009 | 6.87 (±0.71) | $2.81x10^{-13}$ ($\pm 2.93x10^{-14}$) | 22.3 (±0) | 14.8 (±0) |
| 9/14/23 | $NO_2$ (ppb) | PVDF | 1100 (±1.12) | 538.43 (±5.62) | 100 | 103.17 (±0.29) | 101.35 (±0.21) | <0.001 | 3.73 (±1.03) | $1.53x10^{-13}$ ($\pm 4.26x10^{-14}$) | 24.27 (±0.08) | 10.92 (±0.81) |
| 10/26/23 | $NO_2$ (ppb) | PVDF | 1100 (±1.12) | 538.43 (±5.62) | 150 | 155.69 (±0.3) | 153.43 (±0.29) | <0.001 | 4.62 (±1.22) | $1.89x10^{-13}$ ($\pm 5.03x10^{-14}$) | 24 (±N/A) | 14 (±N/A) |

Column descriptions:
F - gas flowrate
SA - thermoplastic baffle surface area
$C_{SP}$ - dilution system gas concentration set point
$C_{A0}$ - baseline concentration measurements without the thermoplastic baffle in the acrylic chamber
$C_{A1}$ - concentration measurements with the thermoplastic baffle in the acrylic chamber
ANOVA p-value - analysis of variance (ANOVA) p-value if the $C_{A0}$ and $C_{A1}$ concentration measurements have different averages. A p-value of <0.05 is considered statistically significant in this study.
$-r_A$ - calculated reaction rate according to Equation 3
Molar $-r_A$ - calculated reaction rate according to Equation 3 converted to molar units
T - average experiment temperature
RH - average experiment relative humidity

# Table S.10

Table S10: Reaction results for volatile organic compounds (VOC) and each of the five thermoplastics - polylactic acid (PLA), acrylonitrile butadiene styrene (ABS), polyethylene terephthalate glycol (PETG), polycarbonate (PC), and polyvinylidene fluoride (PVDF). Two gas concentration tests were run for each of the five thermoplastic materials. A third concentration was not run since both of the initial VOC concentration tests indicate off-gassing instead of reaction with each thermoplastic material.

| Date | Gas (Unit) | Material | F (±std) | SA (±std) | $C_{SP}$ | $C_{A0}$ (±std) | $C_{A1}$ (±std) | ANOVA p-value | $-r_A$ (±error) | Molar$-r_A$ (±error) | T (±std) | RH (±std) |
|---|---|---|---|---|---|---|---|---|---|---|---|---|
| | | | (ccm) | (cm$^2$) | $(ppb)$ | $(ppb)$ | $(ppb)$ | | $\left(\frac{ccm*(ppb)}{cm^2}\right)$ | $\left(\frac{mol}{cm^2*min}\right)$ | (°C) | (%) |
| 7/15/23 | VOC (ppb) | PLA | 1100 (±1.12) | 538.43 (±2.81) | 800 | 905.5 (±5.82) | 949.61 (±2.15) | <0.001 | -90.13 (±16.3) | $-3.69x10^{-12}$ $(\pm 6.68x10^{-13})$ | 24.71 (±0.03) | 10.39 (±0.3) |
| 9/5/23 | VOC (ppb) | PLA | 1100 (±1.12) | 538.43 (±2.81) | 400 | 454.55 (±5.06) | 505.38 (±5.18) | <0.001 | -103.83 (±20.95) | $-4.25x10^{-12}$ $(\pm 8.59x10^{-13})$ | 24 (±0) | 13.26 (±0.12) |
| 7/15/23 | VOC (ppb) | ABS | 1100 (±1.12) | 538.43 (±2.81) | 800 | 908.5 (±5.8) | 953.9 (±4.89) | <0.001 | -92.76 (±21.84) | $-3.80x10^{-12}$ $(\pm 8.95x10^{-13})$ | 24.62 (±0.04) | 9.89 (±0.29) |
| 1/26/23 | VOC (ppb) | ABS | 1100 (±1.12) | 538.43 (±2.81) | 400 | 460 (±0) | 460.35 (±94.72) | <0.001 | -0.07 (±1.48) | $-2.97^{-15}$ $(\pm 6.07x10^{-14})$ | 22.4(±0) | 23.3 (±0) |
| 7/15/23 | VOC (ppb) | PETG | 1100 (±1.12) | 538.43 (±2.81) | 800 | 911.8 (±5.8) | 956.33 (±4.83) | <0.001 | -90.98 (±21.72) | $-3.73x10^{-12}$ $(\pm 8.90x10^{-13})$ | 24.71 (±0.06) | 10.65 (±0.28) |
| 9/5/23 | VOC (ppb) | PETG | 1100 (±1.12) | 538.43 (±2.81) | 400 | 455.68 (±5.06) | 490.85 (±8.8) | <0.001 | -71.84 (±28.34) | $-2.94x10^{-12}$ $(\pm 1.16x10^{-12})$ | 23.75 (±0.07) | 12.89 (±0.25) |
| 7/15/23 | VOC (ppb) | PC | 1100 (±1.12) | 538.43 (±5.62) | 800 | 888.5 (±5.82) | 941.47 (±7.26) | <0.001 | -108.22 (±26.77) | $-4.44x10^{-12}$ $(\pm 1.10x10^{-12})$ | 24.58 (±0.03) | 9.22 (±0.24) |
| 9/5/23 | VOC (ppb) | PC | 1100 (±1.12) | 538.43 (±5.62) | 400 | 454.41 (±5.03) | 474.13 (±8.04) | <0.001 | -40.28 (±26.74) | $-1.65x10^{-12}$ $(\pm 1.10x10^{-12})$ | 23.74 (±0.05) | 12.42 (±0.24) |
| 7/15/23 | VOC (ppb) | PVDF | 1100 (±1.12) | 538.43 (±5.62) | 800 | 903.42 (±5.66) | 935.14 (±10.03) | <0.001 | -64.8 (±32.08) | $-2.66x10^{-12}$ $(\pm 1.32x10^{-12})$ | 24.71 (±0.03) | 8.27 (±0.18) |
| 9/5/23 | VOC (ppb) | PVDF | 1100 (±1.12) | 538.43 (±5.62) | 400 | 451.36 (±6.96) | 465.21 (±11.87) | <0.001 | -28.3 (±38.49) | $-1.16x10^{-12}$ $(\pm 1.58x10^{-12})$ | 23.89 (±0.06) | 13.01 (±6.51) |

Column descriptions:
F - gas flowrate
SA - thermoplastic baffle surface area
$C_{SP}$ - dilution system gas concentration set point
$C_{A0}$ - baseline concentration measurements without the thermoplastic baffle in the acrylic chamber
$C_{A1}$ - concentration measurements with the thermoplastic baffle in the acrylic chamber
ANOVA p-value - analysis of variance (ANOVA) p-value if the $C_{A0}$ and $C_{A1}$ concentration measurements have different averages. A p-value of <0.05 is considered statistically significant in this study.
$-r_A$ - calculated reaction rate according to Equation 3
Molar $-r_A$ - calculated reaction rate according to Equation 3 converted to molar units
T - average experiment temperature
RH - average experiment relative humidity

# Table S.11

Table S11: Sgnificance testing results for VOC off-gassing rates between each of the thermoplastics - polylactic acid (PLA), acrylonitrile butadiene styrene (ABS), polyethylene terephthalate glycol (PETG), polycarbonate (PC), and polyvinylidene fluoride (PVDF).

| Materials | p-value | Adjusted $\alpha$[1] |
|---|---|---|
| PLA and ABS | 0.0001 | 0.0025 |
| PLA and PETG | 0.4419 | 0.0025 |
| PLA and PC | 0.1078 | 0.0025 |
| PLA and PVDF | $8.422\text{x}10^{-6}$ | 0.0025 |
| ABS and PLA | 0.0018 | 0.0025 |
| ABS and PETG | 0.0042 | 0.0025 |
| ABS and PC | 0.0123 | 0.0025 |
| ABS and PVDF | $2.169\text{x}10^{-6}$ | 0.0025 |
| PETG and PLA | 0.3497 | 0.0025 |
| PETG and ABS | $8.722\text{x}10^{-5}$ | 0.0025 |
| PETG and PC | 0.2596 | 0.0025 |
| PETG and PVDF | $7.470\text{x}10^{-7}$ | 0.0025 |
| PC and PLA | 0.1589 | 0.0025 |
| PC and ABS | 0.0038 | 0.0025 |
| PC and PETG | 0.4205 | 0.0025 |
| PC and PVDF | $6.565\text{x}10^{-6}$ | 0.0025 |
| PVDF and PLA | $1.956\text{x}10^{-5}$ | 0.0025 |
| PVDF and ABS | $1.89\text{x}10^{-8}$ | 0.0025 |
| PVDF and PETG | $9.321\text{x}10^{-6}$ | 0.0025 |
| PVDF and PC | $4.062\text{x}10^{-6}$ | 0.0025 |

[1] Bonferoni correction

# Table S.12

Table S12: Nitrogen monoxide (NO) and nitrogen dioxide ($NO_2$) kinetic equation, Equation 2, nonlinear fit parameters.

| Gas (Unit) | Material | k [CI] | $\alpha$ [CI] |
|---|---|---|---|
| | | $[ccm * ppb/cm^2 * ppb^\alpha]$ | [-] |
| NO (ppb) | PLA | $1.24x10^{-3}$ $[8.24x10^{-4}, 1.63x10^{-3}]$ | 1.28 [1.23, 1.32] |
| NO (ppb) | ABS | $8.52x10^{-4}$ $[-5.21x10^{-4}, 2.23x10^{-3}]$ | 1.35 [1.13, 1.58] |
| NO (ppb) | PETG | $6.88x10^{-3}$ $[-4.52x10^{-3}, 1.83x10^{-2}]$ | 1.03 [0.79, 1.26] |
| NO (ppb) | PC | $9.35x10^{-4}$ $[4.75x10^{-4}, 1.39x10^{-3}]$ | 1.22 [1.15, 1.29] |
| NO (ppb) | PVDF | $4.63x10^{-3}$ $[-1.17x10^{-2}, 2.10x10^{-2}]$ | 1.03 [0.51, 1.54] |
| $NO_2$ (ppb) | PLA | $4.31x10^{-2}$ $[1.89x10^{-2}, 6.74x10^{-2}]$ | 0.87 [0.75, 0.98] |
| $NO_2$ (ppb) | ABS | $8.10x10^{-3}$ $[6.19x10^{-3}, 1.00x10^{-2}]$ | 1.35 [1.30, 1.39] |
| $NO_2$ (ppb) | PETG | $1.61x10^{-4}$ $[6.71x10^{-5}, 3.00x10^{-4}]$ | 2.06 [1.76, 2.37] |
| $NO_2$ (ppb) | PC | $1.51x10^{-3}$ $[-1.47x10^{-2}, 1.77x10^{-2}]$ | 1.61 [-0.48, 3.69] |
| $NO_2$ (ppb) | PVDF | $4.02x10^{-2}$ $[-5.34x10^{-2}, 1.34x10^{-1}]$ | 0.96 [0.51, 1.41] |

# Table S.13

Table S13: Nitrogen monoxide (NO) and nitrogen dioxide ($NO_2$) kinetic equation, Equation 2, nonlinear fit parameters converted to molar units.

| Gas (Unit) | Material | $k_1$ [CI][1] | $\alpha$ [CI][2] |
|---|---|---|---|
| | | $\left[\frac{mol}{cm^2 * min * mol^{\alpha}}\right]$ | [-] |
| NO (ppb) | PLA | $5.06x10^{-17}$ [$3.45x10^{-17}$, $6.68x10^{-17}$] | 1.28 [1.23, 1.32] |
| NO (ppb) | ABS | $3.49x10^{-17}$ [$-2.14x10^{-17}$, $9.12x10^{-17}$] | 1.35 [1.13, 1.58] |
| NO (ppb) | PETG | $2.82x10^{-16}$ [$-1.85x10^{-16}$, $7.49x10^{-16}$] | 1.03 [0.79, 1.26] |
| NO (ppb) | PC | $3.83x10^{-17}$ [$1.95x10^{-17}$, $5.71x10^{-17}$] | 1.22 [1.15, 1.29] |
| NO (ppb) | PVDF | $1.90x10^{-16}$ [$-4.85x10^{-16}$, $8.61x10^{-16}$] | 1.03 [0.51, 1.54] |
| $NO_2$ (ppb) | PLA | $1.77x10^{-15}$ [$7.71x10^{-16}$, $2.76x10^{-15}$] | 0.87 [0.75, 0.98] |
| $NO_2$ (ppb) | ABS | $3.32x10^{-16}$ [$2.54x10^{-16}$, $4.10x10^{-16}$] | 1.35 [1.30, 1.39] |
| $NO_2$ (ppb) | PETG | $4.75x10^{-18}$ [$-2.75x10^{-18}$, $1.23x10^{-17}$] | 2.06 [1.76, 2.37] |
| $NO_2$ (ppb) | PC | $6.20x10^{-17}$ [$-6.03x10^{-16}$, $7.27x10^{-16}$] | 1.61 [-0.48, 3.69] |
| $NO_2$ (ppb) | PVDF | $1.65x10^{-16}$ [$-2.19x10^{-15}$, $5.48x10^{-15}$] | 0.96 [0.51, 1.41] |

[1] The optimal reaction rate constant parameter for the molar nonlinear fit is the same as the regular optimal reaction rate constant parameters except that it includes the conversion factors to go from $ccm * ppb$ to $mol$. $-r_A = \frac{k}{(10^9)(24.4)(1000)} C_A^{\alpha} = k_1 C_A^{\alpha}$.

[2] The optimal reaction order parameter for the molar nonlinear fit is the same as the regular optimal reaction order parameter.

# Table S.14

Table S14: FDM thermoplastic housing component impact on trace gas concentrations for VOC, NO, and $NO_2$ in a 2800 $cm^3$ enclosure with a fan that draws air through the housing at a rate of 83,333 ccm resulting in a 2-second gas residence time or 2,222 ccm resulting in a gas residence time of 75 seconds. Using FDM-printed thermoplastic inserts with a total surface area of 118 $cm^2$.

| Gas | Material | Concentration[1] | $\alpha$ | $k$ | $-r_A$ | Range of Flow Rates | Surface Area | Range in Amount of Gas Off-gassed \| Reacted Range | Range in Percent Difference of Concentration |
|---|---|---|---|---|---|---|---|---|---|
| | | (ppb) | (-) | $\left(\frac{ccm*ppb}{cm^2*ppb^\alpha}\right)$ | $\left(\frac{ccm*ppb}{cm^2}\right)$ | (ccm) | ($cm^2$) | (ppb) | |
| VOC | PLA | 41 | N/A | N/A | -18.29 | 2,222 to 83,333 | 118 | $-2.59x10^{-2}$ to $-9.71x10^{-1}$ | $6.31x10^{-2}$ to 2.37 % |
| VOC | ABS | 41 | N/A | N/A | -30.74 | 2,222 to 83,333 | 118 | $-4.35x10^{-2}$ to $-1.63$ | $1.06x10^{-1}$ to 3.98 % |
| VOC | PETG | 41 | N/A | N/A | -19.88 | 2,222 to 83,333 | 118 | $-2.81x10^{-2}$ to $-1.06$ | $6.87x10^{-2}$ to 2.57 % |
| VOC | PC | 41 | N/A | N/A | -21.83 | 2,222 to 83,333 | 118 | $-3.09x10^{-2}$ to $-1.16$ | $7.54x10^{-2}$ to 2.83 % |
| VOC | PVDF | 41 | N/A | N/A | -0.41 | 2,222 to 83,333 | 118 | $-5.78x10^{-4}$ to $-2.17x10^{-2}$ | $1.41x10^{-3}$ to $5.29x10^{-2}$ % |
| NO | PLA | 35 | 1.28 | $1.24x10^{-3}$ | 0.117 | 2,222 to 83,333 | 118 | $1.65x10^{-4}$ to $6.19x10^{3}$ | $4.71x10^{-4}$ to $1.77x10^{-2}$ % |
| NO | ABS | 35 | 1.35 | $8.52x10^{-4}$ | 0.105 | 2,222 to 83,333 | 118 | $1.48x10^{-4}$ to $5.55x10^{-3}$ | $4.23x10^{-4}$ to $1.59x10^{-2}$ % |
| NO | PETG | 35 | 1.03 | $6.88x10^{-3}$ | 0.266 | 2,222 to 83,333 | 118 | $3.77x10^{-4}$ to $1.41x10^{-2}$ | $1.08x10^{-3}$ to $4.03x10^{-3}$ % |
| NO | PC | 35 | 1.22 | $9.35x10^{-4}$ | 0.072 | 2,222 to 83,333 | 118 | $1.01x10^{-4}$ to $3.80x10^{-3}$ | $2.89x10^{-4}$ to $1.09x10^{-2}$ % |
| NO | PVDF | 35 | 1.03 | $4.63x10^{-3}$ | 0.178 | 2,222 to 83,333 | 118 | $2.53x10^{-4}$ to $9.47x10^{-3}$ | $7.22x10^{-4}$ to $2.71x10^{-2}$ % |
| $NO_2$ | PLA | 35 | 0.87 | $4.31x10^{-2}$ | 0.944 | 2,222 to 83,333 | 118 | $2.13x10^{-3}$ to $7.98x10^{-2}$ | $6.08x10^{-3}$ to $2.28x10^{-1}$ % |
| $NO_2$ | ABS | 35 | 1.35 | $8.10x10^{-3}$ | 0.966 | 2,222 to 83,333 | 118 | $1.41x10^{-5}$ to $5.28x10^{-4}$ | $4.02x10^{-5}$ to $1.51x10^{-3}$ % |
| $NO_2$ | PETG | 35 | 2.06 | $1.16x10^{-4}$ | 0.177 | 2,222 to 83,333 | 118 | $1.58x10^{-4}$ to $5.94x10^{-3}$ | $4.52x10^{-4}$ to $1.70x10^{-2}$ % |
| $NO_2$ | PC | 35 | 1.61 | $1.51x10^{-3}$ | 0.456 | 2,222 to 83,333 | 118 | $6.46x10^{-4}$ to $2.42x10^{-2}$ | $1.85x10^{-3}$ to $6.92x10^{-2}$ % |
| $NO_2$ | PVDF | 35 | 0.96 | $4.02x10^{-2}$ | 1.23 | 2,222 to 83,333 | 118 | $9.45x10^{-4}$ to $3.54x10^{-2}$ | $2.70x10^{-3}$ to $1.01x10^{-1}$ % |

[1] Preliminary work for the planned study showed an average VOC concentration of 40 ppb with a max of 1000 ppb from roadside measurements of some idling vehicles. Liu et al. [5] measured vehicle emission in parking garages and found the peak hourly averaged NOx measurement of approximately 35 ppb at the center of the parking garage and 10 ppb at the gate of the parking garage. Therefore, a 40 ppb VOC concentration and 35 ppb NO/NO2 concentrations were used as the theoretical concentrations to estimate the impact of the FDM-printed thermoplastic structural supports in this theoretical exercise.

Column descriptions:

$\alpha$ - reaction experiment result nonlinear fit parameter for reaction order

$k$ - reaction experiment result nonlinear fit parameter for reaction rate constant

$-r_A$ - For NO and $NO_2$, calculated reaction rate according to Equation 2. For VOC, estimated off-gassing rate from off-gassing experiment results.

# Table S.15

Table S15: Worst case FDM thermoplastic housing component impact on trace gas concentrations for VOC, NO, and $NO_2$. This scenario includes lower VOC concentrations but higher NO and $NO_2$ concentrations. With a larger thermoplastic surface area assuming both the 2800 $cm^3$ enclosure (1360 $cm^2$ surface area) and the inserts (118 $cm^2$) are FDM-printed thermoplastics.

| Gas | Material | Concentration | $\alpha$ | $k$ | $-r_A$ | Range of Flow Rates[1] | Surface Area | Range in Amount of Gas Off-gassed \| Reacted Range | Range in Percent Difference of Concentration |
|---|---|---|---|---|---|---|---|---|---|
| | | (ppb) | (-) | $\left(\frac{ccm*ppb}{cm^2*ppb^\alpha}\right)$ | $\left(\frac{ccm*ppb}{cm^2}\right)$ | (ccm) | ($cm^2$) | (ppb) | |
| VOC | PLA | 10 | N/A | N/A | -18.29 | 2,222 to 83,333 | 1478 | -0.324 to -12.2 | 3.24 to 122 % |
| VOC | ABS | 10 | N/A | N/A | -30.74 | 2,222 to 83,333 | 1478 | -0.545 to -20.4 | 5.45 to 204 % |
| VOC | PETG | 10 | N/A | N/A | -19.88 | 2,222 to 83,333 | 1478 | -0.353 to -13.2 | 3.53 to 132 % |
| VOC | PC | 10 | N/A | N/A | -21.83 | 2,222 to 83,333 | 1478 | -0.387 to -14.5 | 3.87 to 145 % |
| VOC | PVDF | 10 | N/A | N/A | -0.41 | 2,222 to 83,333 | 1478 | $-7.25x10^{-3}$ to -0.272 | $7.25x10^{-2}$ to 2.72 % |
| NO | PLA | 600 | 1.28 | $1.24x10^{-3}$ | 4.41 | 2,222 to 83,333 | 1478 | $7.83x10^{-2}$ to 7.05 | $1.30x10^{-2}$ to 1.17 % |
| NO | ABS | 600 | 1.35 | $8.52x10^{-4}$ | 4.88 | 2,222 to 83,333 | 1478 | $8.66x10^{-2}$ to 7.79 | $1.44x10^{-2}$ to 1.3 % |
| NO | PETG | 600 | 1.03 | $6.88x10^{-3}$ | 4.94 | 2,222 to 83,333 | 1478 | $8.76x10^{-2}$ to 7.88 | $1.46x10^{-2}$ to 1.31 % |
| NO | PC | 600 | 1.22 | $9.35x10^{-4}$ | 2.29 | 2,222 to 83,333 | 1478 | $4.07x10^{-2}$ to 3.66 | $6.78x10^{-3}$ to 0.61 % |
| NO | PVDF | 600 | 1.03 | $4.63x10^{-3}$ | 3.30 | 2,222 to 83,333 | 1478 | $5.86x10^{-2}$ to 5.27 | $9.77x10^{-3}$ to 0.879 % |
| $NO_2$ | PLA | 200 | 0.87 | $4.31x10^{-2}$ | 4.29 | 2,222 to 83,333 | 1478 | $7.60x10^{-2}$ to 2.85 | $3.80x10^{-2}$ to 1.43 % |
| $NO_2$ | ABS | 200 | 1.35 | $8.10x10^{-3}$ | 10.1 | 2,222 to 83,333 | 1478 | 0.179 to 6.70 | $8.94x10^{-2}$ to 3.35 % |
| $NO_2$ | PETG | 200 | 2.06 | $1.16x10^{-4}$ | 6.46 | 2,222 to 83,333 | 1478 | 0.115 to 4.29 | $5.73x10^{-2}$ to 2.15 % |
| $NO_2$ | PC | 200 | 1.61 | $1.51x10^{-3}$ | 7.49 | 2,222 to 83,333 | 1478 | 0.113 to 4.98 | $6.64x10^{-2}$ to 2.49 % |
| $NO_2$ | PVDF | 200 | 0.96 | $4.02x10^{-2}$ | 6.60 | 2,222 to 83,333 | 1478 | 0.117 to 4.39 | $5.85x10^{-2}$ to 2.19 % |

[1] For a sensor housing with a volume of 2800 $cm^3$, a flow rate of 2,222.2 ccm produces a gas residence time of 75 seconds, while a flow rate of 83,333.3 ccm produces a gas residence time of 2 seconds.

Column descriptions:

$\alpha$ - reaction experiment result nonlinear fit parameter for reaction order

$k$ - reaction experiment result nonlinear fit parameter for reaction rate constant

$-r_A$ - For NO and $NO_2$, calculated reaction rate according to Equation 2. For VOC, estimated off-gassing rate from off-gassing experiment results.

# Section S.1: FDM-printed Baffle Surface Area Calculation and Uncertainty

The surface area calculation for the FDM-printed baffle was based on CAD models. Measurements were made within the CAD software, Fusion360, to calculate the expected surface area for the printed baffle. Uncertainty was added to the surface area calculation using the "print precision" obtained from documentation for the FlashForge Creator Pro 2 [2] and LulzBot TAZ 6 [6] printers.

The expression used to calculate the FDM-printed baffle surface area combines the surface area of the base

$$SA_{base} = w_{base}l_{base} + 16w_{out}h_{out} + 16h_{out}l_{out} + 8w_{out}l_{out} \tag{S1}$$

and the surface area of the vertical baffles

$$SA_{baffle} = 2l_{baffle}h_{baffle} + 2l_{bafffle}w_{baffle} + 2w_{baffle}h_{baffle} - 80w_{void}^2 + 32h_{baffle}w_{void} \tag{S2}$$

into the final surface area for the assembled baffle

$$SA = SA_{base} + SA_{baffles} = 538.43cm^2. \tag{S3}$$

Where $SA$ is the calculated surface area of the assembled baffle, $SA_{base}$ and $SA_{baffle}$ are the calculated surface areas of the base and baffles that combine into the baffle assembly, and $l$, $w$, and $h$ are the length, width, and height of either the base or the baffle as annotated. Equation 1 neglects the surfaces of the base that are in contact with the bottom or side walls of the acrylic chamber. Resulting in an assembled baffle surface area of 538.43 cm$^2$.

The uncertainty of the assembled baffle surface area was also calculated by combining the uncertainty in the surface area of the base

$$e_{base} = \sqrt{\sigma^2[(l_{base})^2 + (16h_{out} + 8l_{out})^2 + (w_{base})^2 + (16w_{out} + 16l_{out})^2 + (16h_{out} + 8w_{out})^2]} \tag{S4}$$

and the uncertainty in the surface area of the vertical baffles

$$e_{baffle} = \sqrt{\begin{array}{r}\sigma^2[(2h_{baffle} + 2w_{baffle})^2 + (2l_{baffle} + 2w_{baffle})^2 \\ +(2l_{baffle} + 2w_{baffle} + 32w_{void})^2 + (-160w_{void} + 32h_{baffle})^2]\end{array}} \tag{S5}$$

into the final uncertainty in the surface area for the assembled baffle

$$e_{SA} = e_{base} + e_{vertical} = 2.81cm^2(FlashForge)\ or\ 5.62cm^2(LulzBot). \tag{S6}$$

Where $e_{SA}$ is the calculated uncertainty for the assembled baffle surface area, $e_{base}$ and $e_{baffle}$ are the calculated uncertainties for the base surface area and baffle surface, $\sigma$ is the "print precision" for either the FlashForge Creator 2 Pro or the LulzBot TAZ6, and $l$, $w$, and $h$ are the length, width, and height of either the base or the baffle as annotated. Resulting in an assembled baffle surface area uncertainty of 2.81 cm$^2$ for the FlashForge Creator 2 Pro and 5.62 cm$^2$ for the LulzBot TAZ6.

# Section S.2: Volumetric Flow Rate Uncertainty Calculation

Uncertainty in the dilution system total volumetric flow rate is determined based on the standard deviation of measured volumetric flow rates for each mass flow controller (MFC). The volumetric flow rate error is calculated by summing the standard deviations for each MFC

$$e_F = \sigma_{MFC_1} + \sigma_{MFC_2} + \sigma_{MFC_3} = 1.12ccm. \tag{S7}$$

Where $e_F$ is the uncertainty in the volumetric flow rate, $\sigma_{MFC_\#}$ is the standard deviation of the measured volumetric flow rates for each MFC.

## Section S.3: Equipment List

- NO and $NO_2$ Reference sensor: ThermoFisher NOx Analyzer Model 42i Part Number: 101350-00
- CO and $CO_2$ Reference sensor: TSI Q-TRAK Indoor Air Quality Monitor Model 7575 with Probe 982
- VOC Reference sensor: TSI Q-TRAK Indoor Air Quality Monitor Model 7575 with Probe 984
- Zero Air Generator: Teledyne High-Performance Zero Air Generator-Model T701H
- Mass Flow Controllers: MasterFlex Model Number 32907-67
- Temperature and Relative Humidity sensor: Aosong DHT22
- Microcontroller: Arduino Nano ATmega328
- High Temperature 3D printer: LulzBot TAZ 6
- 3D printer: FlashForge Creator Pro 2 EN-AO1
- Filaments:
  - ABS: HATCHBOX Model Number-3D ABS-1KG1.75-WHT. CAS number: 9003-56-9
  - PC: PolyMaker Polylite PC Model Number-PC01001. CAS number: 25037-45-0
  - PETG: HATCHBOX Model Number-3D PETG-1KG1.75-BLK. CAS number: 25640-14-6
  - PLA: HATCHBOX Model Number-3D PLA-1KG1.75-WHT. CAS number: 26100-51-6
  - PVDF: FLOURX Model Number-PVD01UN100750NAT0. CAS number: 24937-79-9
- Gasses:
  - Zero Air: AirGas Part Number: UN1002
  - Carbon monoxide: GASCO Part Number: 103L-50-500
  - Carbon dioxide: AirGas Part Number: UN1956
  - Nitrogen monoxide: GASCO Part Number: 116L-125-20
  - Nitrogen dioxide: GASCO Part Number: 116L-111-10
  - Isobutylene(for VOC reaction experiments): MESA Part Number: U105520PA

## Section S.4: VOC Off-gassing Results Null Hypothesis Significance Testing

A null hypothesis significance test (NHST) was conducted to determine if there was a statistically significant difference between VOC off-gassing experiment reaction rates among each of the thermoplastics. The reaction rate result for each thermoplastic is a single calculated value with a single calculated estimated error value that were used to calculate the t-statistic for the NHST. The null hypothesis was defined as:

$$H_0 : -r_{A_{plasticX}} = -r_{A_{plasticY}}, \tag{S8}$$

and the t-statistic was calculated using:

$$t_{statistic} = \frac{-r_{A_{plasticX}} - (-r_{A_{plasticY}})}{e_{-r_{A_{plasticX}}} / \sqrt{N}}. \tag{S9}$$

Where $-r_{A_{plasticX}}$ and $-r_{A_{plasticY}}$ are the reaction rates for the thermoplastics that are being compared; $e_{-r_{A_{plasticX}}}$ is the estimated error in the reaction rate; and $N$ is the number of concentration measurements used to calculate the reaction rate.

The t-statistic was converted to a p-value using the survival function, "scipy.stats.t.sf," with degrees of freedom as an input argument to the method. Additionally, a Bonferoni correction was applied to create an adjusted significance level ($\alpha_{adjusted}$) since a total of 20 NHST were conducted at once between each thermoplastic.

$$\alpha_{adjusted} = \frac{0.05}{20} = 0.0025 \tag{S10}$$

Table S11 lists the results for the VOC off-gassing NHST.

## Section S.5: FDM-printed Baffle Design and FDM Printer Settings

The FDM-printed baffles were designed in Fusion360 computer aided design software. The baffle structure was designed in two pieces: a 9.3 cm x 11.5 cm x 0.15 cm base plate and four 6.5 cm x 5.8 cm x 0.15 cm vertical baffles. Each vertical baffle was covered with 80 evenly spaced 0.4 cm x 0.4 cm holes. The base plate design included inserts to mount the four vertical baffles, with two baffles aligned at 20-degree inward angles on each side. Figure S.S3 shows a screenshot of the base plate created in Fusion360. Figure S.S4 shows a screenshot of the vertical baffles created in Fusion360. The final baffle assembly was created by sliding the vertical baffles into the inserts of the base plate.

FlashPrint 5 slicing software was used for the baffles printed using the FlashForge Creator Pro 2 printer, while Cura LulzBot edition slicing software was used for the LulzBot TAZ6 printer. Both FDM printers, FlashForge Creator Pro 2 and LulzBot TAZ6, were set to print the baffles with a 15% infill and four layers for the shell for each thermoplastic. Additional settings for each thermoplastic were:

- ABS (FlashForge Creator Pro 2)
  - Extrusion temperature: 240°C
  - Bed temperature: 50°C
  - All other print parameters were left as default.
- PLA (FlashForge Creator Pro 2)
  - Extrusion temperature: 240°C
  - Bed temperature: 50°C
  - All other print parameters were left as default.
- PETG (FlashForge Creator Pro 2)
  - Extrusion temperature: 240°C
  - Bed temperature: 50°C
  - All other print parameters were left as default.
- PC (LulzBot TAZ6)
  - Extrusion temperature: 280°C
  - Bed temperature: 110°C
  - All other print parameters were left as default.
- PVDF (LulzBot TAZ6)
  - Extrusion temperature: 280°C
  - Bed temperature: 110°C
  - All other print parameters were left as default.
  - When printing with PVDF filament, the Lulzbot TAZ6 printer was placed inside a gas hood due to possible fluorine emissions [7].

# Section S.6: Supplementary Material References

## References


[1] Rashid Dallaev et al. "Brief Review of PVDF Properties and Applications Potential". In: *Polymers* 14 (22 Nov. 2022). ISSN: 20734360. DOI: 10.3390/polym14224793.

[2] FlashForge. *Creator Pro 2 Specification.* https://www.flashforge.com/product-detail/flashforge-creator-pro-2-3d-printer [Accessed: 12/22/2023].

[3] R. Hagen. "Polylactic Acid". In: vol. 10. Elsevier, 2012, pp. 231–236. ISBN: 9780080878621. DOI: 10.1016/B978-0-444-53349-4.00269-7.

[4] Matthew R. Hartings and Zeeshan Ahmed. "Chemistry from 3D printed objects". In: *Nature Reviews Chemistry* 3 (5 May 2019), pp. 305–314. ISSN: 23973358. DOI: 10.1038/s41570-019-0097-z. URL: https://doi.org/10.1038/s41570-019-0097-z.

[5] Bingqi Liu and Naomi Zimmerman. "Fleet-based vehicle emission factors using low-cost sensors: Case study in parking garages". In: *Transportation Research Part D: Transport and Environment* 91 (Feb. 2021), p. 102635. ISSN: 13619209. DOI: 10.1016/j.trd.2020.102635. URL: https://www.sciencedirect.com/science/article/pii/S1361920920308208?via%3Dihub.

[6] Lulzbot. *Lulzbot Taz6 Desktop 3D Printer Complete Technical Specifications.* https://lulzbot.com/store/taz-6 [Accessed: 12/22/2023].

[7] 3DXTECH Additive Manufacturing. *Safety Data Sheet FLUORX$^{TM}$ PVDF [POLYVINYLIDINE FLUORIDE POLYMER] Revision V1.1.* https://www.3dxtech.com/tech-data-sheets-safety-data-sheets/ [Accessed: 12/22/2023]. Aug. 2022.

[8] Laurence W. McKeen. "Styrenic Plastics". In: Elsevier, 2010, pp. 51–71. DOI: 10.1016/b978-0-08-096450-8.00004-1.

[9] Durgam Muralidharan Nivedhitha and Subramanian Jeyanthi. "Polyvinylidene fluoride, an advanced futuristic smart polymer material: A comprehensive review". In: *Polymers for Advanced Technologies* 34 (2 2023), pp. 474–505. ISSN: 10991581. DOI: 10.1002/pat.5914.

[10] Georgii B Pariiskii, I S Gaponova, and Evgenii Ya Davydov. "Reactions of nitrogen oxides with polymers". In: *Russian Chemical Reviews* 69 (11 Nov. 2000), pp. 985–999. ISSN: 0036-021X. DOI: 10.1070/rc2000v069n11abeh000611.

[11] Lalit Ranakoti et al. "Critical Review on Polylactic Acid: Properties, Structure, Processing, Biocomposites, and Nanocomposites". In: *Materials* 15 (12 June 2022). ISSN: 19961944. DOI: 10.3390/ma15124312. URL: https://doi.org/10.3390/ma15124312.

[12] K. Takeuchi. "5.16 - Polycarbonates". In: vol. 5. Elsevier, Jan. 2012, pp. 363–376. ISBN: 9780080878621. DOI: 10.1016/B978-0-444-53349-4.00148-5.

[13] Irina Turku, Sushil Kasala, and Timo Kärki. "Characterization of Feedstock Filament Extruded from Secondary Sources of PS, ABS and PVC". In: 2018. URL: https://api.semanticscholar.org/CorpusID:53317722.

[14] S. R. Turner and Y. Liu. "5.14 - Chemistry and Technology of Step-Growth Polyesters". In: vol. 5. Elsevier, Jan. 2012, pp. 311–331. ISBN: 9780080878621. DOI: 10.1016/B978-0-444-53349-4.00143-6.

[15] Yingshuang Zhang et al. "Flotation separation of acrylonitrile-butadiene-styrene and polystyrene in WEEE based on oxidation of active sites". In: *Minerals Engineering* 146 (Jan. 2020). ISSN: 08926875. DOI: 10.1016/j.mineng.2019.106131. URL: https://doi.org/10.1016/j.mineng.2019.106131.
